# Reappraising host cellular factors involved in attachment and entry to develop antiviral strategies against porcine reproductive and respiratory syndrome virus

**DOI:** 10.3389/fmicb.2022.975610

**Published:** 2022-07-26

**Authors:** Rui Li, Songlin Qiao, Gaiping Zhang

**Affiliations:** Key Laboratory of Animal Immunology of the Ministry of Agriculture, Henan Provincial Key Laboratory of Animal Immunology, Henan Academy of Agricultural Sciences, Zhengzhou, China

**Keywords:** PRRSV, attachment, entry, host cellular factors, antiviral strategies

## Abstract

Porcine reproductive and respiratory syndrome (PRRS), caused by PRRS virus (PRRSV), is a highly contagious disease that brings tremendous economic losses to the global swine industry. As an intracellular obligate pathogen, PRRSV infects specific host cells to complete its replication cycle. PRRSV attachment to and entry into host cells are the first steps to initiate the replication cycle and involve multiple host cellular factors. In this review, we recapitulated recent advances on host cellular factors involved in PRRSV attachment and entry, and reappraised their functions in these two stages, which will deepen the understanding of PRRSV infection and provide insights to develop promising antiviral strategies against the virus.

## Introduction

As intracellular obligate pathogens, viruses infect host cells to complete their replication cycles, including attachment, entry, replication, synthesis, assembly, and release (Jones et al., 2021). Attachment and entry are the first and essential steps for viruses to establish infection, which are ideal antiviral targets (Lu et al., 2021; Tompa et al., 2021). Therefore, an in-depth investigation of viral attachment and entry will provide novel insights to develop potent antiviral strategies.

Porcine reproductive and respiratory syndrome (PRRS) is a highly contagious swine disease characterized by reproductive failures in sows of late-term gestation and respiratory diseases in pigs of all ages (Done and Paton, 1995; Rossow, 1998). Since its emergence in the late 1980s, PRRS keeps burdening the global swine industry with an annual economic loss of up to $664 million in the United States (Holtkamp et al., 2013). PRRS virus (PRRSV), as the causative agent, is an enveloped single-stranded positive-sense RNA virus and belongs to the order *Nidovirales*, family *Arteriviridae*, and genus *Betaarterivirus*.^1^

Infection by PRRSV shows a strongly restricted tropism for target cells, including porcine alveolar macrophages (PAMs) *in vivo* (Duan et al., 1997), and African green monkey kidney epithelial cell line MA-104 and its derivative MARC-145 *in vitro* (Kim et al., 1993). Multiple host cellular factors have been reported to be involved in PRRSV attachment to and entry into these target cells (Shi et al., 2015; Zhang and Yoo, 2015), such as heparan sulfate (HS) (Delputte et al., 2002), sialoadhesin (Sn/CD169) (Vanderheijden et al., 2003), vimentin (Kim et al., 2006), CD163 (Calvert et al., 2007), CD151 (Shanmukhappa et al., 2007), dendritic cell-specific intercellular adhesion molecule-3-grabbing non-integrin (DC-SIGN/CD209) (Huang et al., 2009).

HS, Sn, and CD163 have been intensively studied during PRRSV infection. According to a previous model (Van Breedam et al., 2010a), PRRSV firstly bound to HS on the target cell surface (Jusa et al., 1997) and subsequently interacted with Sn to be internalized *via* low pH-dependent clathrin-mediated endocytosis (Kreutz and Ackermann, 1996; Nauwynck et al., 1999; Delputte et al., 2005). During internalization, PRRSV was associated with CD163, which was considered to cooperate with Sn in facilitating viral internalization (Van Gorp et al., 2008) as well as to mediate viral membrane fusion and uncoating (Yu et al., 2019).

However, this model is challenged based on recent studies of PRRSV infection. For example, Sn knockout pigs are susceptible to PRRSV, demonstrating that it is not required for PRRSV attachment and internalization (Prather et al., 2013). In addition to attachment and entry, HS and vimentin are shown to participate in other stages of PRRSV replication cycle (Song et al., 2016; Guo et al., 2017a; Chang et al., 2018; Zheng et al., 2021). Moreover, several novel host cellular factors are identified to be involved in PRRSV attachment and entry, such as non-muscle myosin heavy chain 9 (MYH9) (Gao et al., 2016), syndecan-4 (Wang et al., 2016a), epidermal growth factor receptor (EGFR) (Wang et al., 2016b), T-cell immunoglobulin and mucin domain (TIM)-1/4 (Wei et al., 2020), and heat shock protein member 8 (HSPA8) (Wang et al., 2022).

In this review, we summarized recent advances on host cellular factors involved in PRRSV attachment and entry, and re-evaluated their roles in PRRSV infection in the hope of supporting ideas for developing antiviral strategies against the virus (Table 1).

**TABLE 1:** Functional reappraisal of and antiviral strategies against host cellular factors involved in PRRSV attachment and entry.

Factor	Function	Antiviral strategies
CD163	Indispensable receptor (Whitworth et al., 2016)	Gene-edited pigs (Whitworth et al., 2016; Wells et al., 2017; Burkard et al., 2018; Yang et al., 2018; Chen et al., 2019; Guo et al., 2019a; Wang et al., 2019a; Xu et al., 2020b) MicroRNAs (Gao et al., 2013; Zhu et al., 2014; Li et al., 2021) The recombinant protein (Chen et al., 2014; Xia et al., 2018) MAbs (Xu et al., 2020a; Zhang et al., 2020d; Han et al., 2022) Peptides (Han et al., 2022) Small molecules (Huang et al., 2020)
Sn	Attachment and entry co-factor? (Prather et al., 2013) Negative regulator of host innate immunity (Liu et al., 2020a; Zhang et al., 2020a)	MAbs (Duan et al., 1998) The recombinant protein (Chen et al., 2014; Xia et al., 2018) MicroRNA (Zhu et al., 2014)
HS	Attachment factor (Delputte et al., 2002) Release factor (Guo et al., 2017a)	Heparin (Delputte et al., 2002) Heparinase (Delputte et al., 2002) Heparanase inhibitor (Guo et al., 2017b)
Vimentin	Attachment factor (Kim et al., 2006; Song et al., 2016) Replication factor (Song et al., 2016; Chang et al., 2018; Zheng et al., 2021) Transport factor (Liang et al., 2020)	Antibodies (Kim et al., 2006) Inhibitors (Zheng et al., 2021)
MYH9	Essential attachment and entry co-factor (Gao et al., 2016) Negative regulator of host innate immunity (Liu et al., 2019) Transport factor (Liang et al., 2020)	Myosin II ATPase inhibitor (Gao et al., 2016) MicroRNA (Li et al., 2016) The recombinant protein (Li et al., 2018b) Disassembly inducer (Xue et al., 2019) Anti-idiotypic antibody (Li et al., 2019)
TIMs	Signaling factor (Wei et al., 2020)	
HSPA8	Attachment and entry co-factor (Wang et al., 2022)	Polyclonal antibodies (Wang et al., 2022) The recombinant protein (Wang et al., 2022) Inhibitors (Wang et al., 2022)
Syndecan -4	Attachment factor (Wang et al., 2016a)	
EGFR	Signaling factor (Wang et al., 2016b)	
Siglec-10	Entry co-factor (Xie et al., 2017)	

## Previously identified host cellular factors

### CD163

CD163 is a member of class I scavenger receptors (SRs) (Law et al., 1993; Zani et al., 2015). It is a type I membrane glycoprotein consisting of nine SR cysteine-rich (SRCR) domains (SRCR1-9) and two proline-serine-threonine (PST)-rich motifs (PST I and II) in its large extracellular region, a single transmembrane domain and a short cytoplasmic tail (Van Gorp et al., 2010a). CD163 plays critical roles under physiological and pathological conditions, such as hemoglobin-haptoglobin SR (Kristiansen et al., 2001), erythroblast adhesion receptor (Fabriek et al., 2007), and TWEAK SR (Bover et al., 2007; Moreno et al., 2009; Akahori et al., 2015). CD163 functions as an innate immune sensor for bacteria (Fabriek et al., 2009) and an anti-inflammatory receptor for HMGB1-haptoglobin complexes (Yang et al., 2016). Moreover, it is a receptor for simian hemorrhagic fever virus (Caì et al., 2015) and PRRSV (Welch and Calvert, 2010).

CD163 expression renders various non-permissive cells susceptible to PRRSV infection (Calvert et al., 2007; Lee et al., 2010; Wang et al., 2013, 2019c; Li et al., 2017; Xu et al., 2020c). CD163 knockout pigs are fully resistant to PRRSV, confirming that it is an indispensable receptor for PRRSV (Whitworth et al., 2016; Yang et al., 2018; Xu et al., 2020b). CD163 SRCR5 domain is further demonstrated to be crucial for PRRSV infection both *in vitro* and *in vivo* (Van Gorp et al., 2010b; Burkard et al., 2017, 2018; Wells et al., 2017; Chen et al., 2019; Guo et al., 2019a; Wang et al., 2019a). Consequently, CD163 is the most suitable target for preventing and controlling PRRS.

First of all, modulation of CD163 expression is effective in restraining PRRSV infection. As described above, gene editing *via* CRISPR-Cas9 has been applied to breed pigs lacking functional CD163, which confers resistance to PRRSV (Prather et al., 2017; Whitworth and Prather, 2017; Tu et al., 2022). However, considering its significant physiological roles, pigs with deletion or partial deletion of CD163 SRCR5 domain are probably superior to those lacking intact CD163 (Reiner, 2016). Down-regulation of CD163 expression by microRNAs also inhibits PRRSV infection *in vitro* (Gao et al., 2013; Zhu et al., 2014; Li et al., 2021). With progress in efficient delivery techniques (Momin et al., 2021), microRNAs targeting CD163 can be utilized as *in vivo* anti-PRRSV reagents. In addition to direct knockout or knockdown, CD163 upstream regulators can be targeted to modulate its expression. For instance, a disintegrin and metalloprotease 17 (ADAM17) down-regulates CD163 expression and hinders PRRSV entry *in vitro* (Guo et al., 2014b; Zhu et al., 2020), and therefore ADAM17 overexpression *via* genetic modification methods may enhance resistance to PRRSV infection *in vivo*.

CD163 itself can be exploited to restrict PRRSV infection. On the one hand, the recombinant adenovirus-delivered soluble CD163 SRCR5-9 protein has been shown to suppress PRRSV infection both *in vitro* and *in vivo* (Chen et al., 2014; Xia et al., 2018). On the other hand, specific monoclonal antibodies (mAbs) targeting CD163 SRCR5, SRCR6, SRCR7, or PST I domain have been recently reported to inhibit PRRSV infection *in vitro* (Xu et al., 2020a; Zhang et al., 2020d; Han et al., 2022). In one study, peptides derived from the epitopes bound by the mAbs display inhibitory effect on PRRSV infection in a dose-dependent manner (Han et al., 2022). Since CD163 does not mutate as quickly as PRRSV, the recombinant CD163 protein along with anti-CD163 mAbs and peptides are promising to be developed as broad-spectrum therapeutic agents against different PRRSV isolates.

The crystal structure of the CD163 SRCR5 domain determined by us greatly facilitates precise control and prevention of PRRS from the receptor perspective (Ma et al., 2017, 2021). Site-directed mutagenesis of the CD163 SRCR5 domain will be beneficial for breeding gene-edited pigs resistant to PRRSV while maintaining CD163 biological functions (Stoian et al., 2022). Based on the crystal structure, a set of small molecule compounds targeting CD163 SRCR5 have been screened through artificial intelligence molecular screening and validated against PRRSV infection *in vitro* (Huang et al., 2020).

### Sialoadhesin

Sn belongs to the sialic acid-binding Ig-like lectin (Siglec) family, namely Siglec-1. It is a macrophage-restricted molecule with an extracellular domain consisting of one N-terminal V-set Ig-like domain and 16 C2-set domains, a transmembrane domain, and a short cytoplasmic tail (Crocker et al., 1994; Hartnell et al., 2001). Sn was first identified as a sialic acid-dependent sheep erythrocyte receptor and subsequently shown to participate in other physiological or pathological processes (O’Neill et al., 2013).

A prepared mAb was characterized to block PRRSV infection in PAMs and afterward identified to recognize Sn (Duan et al., 1998). Sn was further found to take part in PRRSV attachment and internalization *via* interaction with PRRSV glycoprotein (GP) 5 dependent on its sialic acid-binding activity of V-set Ig-like domain *in vitro* (Vanderheijden et al., 2003; Delputte and Nauwynck, 2004; Delputte et al., 2007; An et al., 2010; Van Breedam et al., 2010b,^2013^; Jiang et al., 2013). However, the involvement of Sn in PRRSV attachment and entry is mired in controversy for MA-104 and MARC-145 cell lines permissive to PRRSV possess no Sn, whereas CD163 by itself is capable of mediating PRRSV infection (Kim et al., 1993; Wang et al., 2013). Sn knockout pigs further prove that Sn is dispensable for PRRSV attachment and internalization *in vivo* (Prather et al., 2013).

Interestingly, co-expression of Sn and CD163 in non-permissive cells significantly enhances PRRSV infection compared to the expression of CD163 alone (Van Gorp et al., 2008). Besides, additive anti-PRRSV effects are observed with simultaneous administration of recombinant Sn and CD163 proteins or Sn- and CD163-targeted microRNAs (Chen et al., 2014; Zhu et al., 2014; Xia et al., 2018). These results suggest that Sn may function differently as an indispensable receptor during PRRSV infection.

Sn has been shown to antagonize antiviral immune responses as other Siglecs (Crocker et al., 2007; Zheng et al., 2015; Akiyama et al., 2017). A recent study unravels that Sn suppresses host innate immunity by down-regulating antiviral cytokine production during PRRSV infection (Zhang et al., 2020a). We further elaborate that Sn interacts with the immune adaptor DNAX-activation protein of 12 kDa (DAP12) to attenuate PRRSV-triggered nuclear factor kappa B (NF-κB) activation and negatively regulate host antiviral innate immune (Liu et al., 2020a,b).

Despite its controversial role in PRRSV infection, Sn is still expected to be conducive to PRRSV eradication in pigs because the adenovirus-delivered recombinant Sn protein additively protects pigs from PRRSV along with CD163 (Xia et al., 2018).

### Heparan sulfate

HS is a linear, unbranched, negatively charged polysaccharide attached to various cell surface or extracellular matrix proteins. It mediates cellular signaling, maintains homeostasis, and regulates cellular growth and metabolism (Ling et al., 2022). At first, HS was found bound by PRRSV matrix protein during viral attachment to PAMs (Delputte et al., 2002, 2005). Various viruses exploit HS for attachment to host cells (Agelidis and Shukla, 2020), including severe acute respiratory syndrome virus 2 (SARS-CoV-2) (Clausen et al., 2020; Zhang et al., 2020b; Chu et al., 2021).

As HS usually functions as the first attachment factor to concentrate virus particles on the target cell surface, interference with the interaction between HS and viruses is supposed to be a potential antiviral approach (Cagno et al., 2019; Cheudjeu, 2021). PRRSV treated with heparin or PAMs treated with heparinase resulted in a significant reduction in viral infection (Delputte et al., 2002). One study also shows a strong anti-SARS-CoV-2 activity by heparin (Tandon et al., 2021).

In 2017, Guo et al. (2017a) showed that HS expression on the cell surface was down-regulated by heparanase to facilitate viral release. Heparanase was up-regulated by PRRSV during the late-stage infection, where *heparanase* knockdown suppressed PRRSV release while its overexpression enhanced. Inhibition of heparanase by pyrithione, a zinc ionophore used as an antibacterial and antifungal agent, has been shown to block PRRSV release (Guo et al., 2017b). A recent study also determines heparanase as a potential target for SARS-CoV-2 for a heparanase inhibitor Roneparstat (in phase I clinical trial for multiple myeloma therapy) reduces viral infection (Xiang et al., 2022).

Based on these results, both HS and heparanase can be targeted to interfere with PRRSV attachment and release.

### Vimentin

Vimentin is a major component of class-III intermediate filaments, which stabilizes the cytoskeleton and maintains cell integrity (Goldman et al., 1996). Vimentin was previously shown to bind to PRRSV nucleocapsid (N) protein, and anti-vimentin antibodies were found to block PRRSV infection in MARC-145 cells (Kim et al., 2006). Delivery of the recombinant simian vimentin was further indicated to render non-susceptible cell lines susceptible to PRRSV (Kim et al., 2006). Next, vimentin was identified to form a complex with PRRSV non-structural protein 2 and N protein that may be essential for viral attachment and replication (Song et al., 2016). Afterward, vimentin was revealed to bind to Annexin A2 and contribute to PRRSV multiplication (Chang et al., 2018). We have recently demonstrated that vimentin reorganizes into cage-like structures enwrapping the PRRSV replication complex during the post-entry stage and is beneficial for PRRSV replication *in vitro*. PRRSV replication is significantly lowered by either 3, 3′-iminodipropionitrile to inhibit vimentin dynamics and network or a specific inhibitor KN-93 targeting calcium calmodulin-dependent protein kinase II gamma responsible for vimentin rearrangement (Zheng et al., 2021). Moreover, PRRSV particles are visualized to move along vimentin during intracellular transport (Liang et al., 2020).

Increasing studies corroborate vimentin as a promising antiviral target due to its diverse functions in viral replication cycles (Ramos et al., 2020; Zhang et al., 2020c). Consequently, vimentin is appropriately targeted for developing antibodies and chemical inhibitors against PRRSV since it affects viral attachment, replication, and transport.

## Newly identified host cellular factors

### Non-muscle myosin heavy chain 9

MYH9, also referred to as non-muscle myosin heavy chain IIA (NMHC-IIA), is a subunit of non-muscle myosin IIA (NM-IIA) (Vicente-Manzanares et al., 2009). MYH9 participates in various cellular physiological processes, including cell shape maintenance, adhesion, migration, signal transduction, and division (Heissler and Manstein, 2013; Pecci et al., 2018).

It has been verified that MYH9 is an essential factor for PRRSV infection *via* interaction with PRRSV GP5 dependent on its C-terminal domain by Professor Zhou’s group (Gao et al., 2016). They demonstrate that the MYH9 C-terminal domain interacts with the CD163 SRCR1-4 domains to facilitate PRRSV internalization in permissive cells (Hou et al., 2019). They further define that the MYH9 C-terminal domain was directly bound by the PRRSV GP5 first ectodomain which induces MYH9 aggregation and polymerization required for PRRSV internalization (Xue et al., 2019). Later, the group identified MYH9 key residues involved in PRRSV internalization by a specific anti-idiotypic antibody Mab2-5G2 to the viral GP5 (Li et al., 2019). Based on these results, MYH9 is demonstrated to be essential for PRRSV attachment and entry.

On the other hand, we uncover that MYH9 recognizes sialic acids on PRRSV GP5 and interacts with DAP12 to activate downstream spleen tyrosine kinase (Syk), resulting in antagonized antiviral pro-inflammatory responses. More importantly, the MYH9-DAP12-Syk pathway plays a negative regulatory role in pro-inflammatory responses upon recognizing sialylated RNA viruses or sialic acid mimics (Liu et al., 2019).

As mentioned above, PRRSV particles also contact MYH9 during intracellular transport along with other cytoskeleton components (Liang et al., 2020).

These extensive studies on MYH9 are helpful for the development of control strategies against PRRSV. The specific myosin II ATPase inhibitor blebbistatin inhibits PRRSV infection *in vitro* and *in vivo* demonstrated by Professor Zhou’s group (Gao et al., 2016). MicroRNA let-7f-5p is reported to significantly suppress PRRSV replication by lowering MYH9 expression (Li et al., 2016). Their data also show that pre-incubation of PRRSV with the MYH9 C-terminal domain suppresses viral infection in susceptible cells in a dose-dependent manner, suggesting that it may serve as a novel anti-PRRSV agent *in vivo* (Li et al., 2018b). Moreover, overexpression of MYH9-specific disassembly inducer S100A4 remarkably leads to diminished MYH9 aggregation and decreased PRRSV internalization in MARC-145 cells (Xue et al., 2019). The anti-idiotypic antibody Mab2-5G2 is demonstrated to diminish PRRSV internalization in PAMs *via* interruption of direct interaction between MYH9 and GP5, which may act as another antiviral agent against the virus in pigs (Li et al., 2019). Our finding of MYH9 as a negative regulator of inflammation provides a molecular basis to design anti-inflammatory drugs against highly pathogenic (HP)-PRRSV (Liu et al., 2019), which causes aberrant pro-inflammatory responses, high fever, morbidity, and mortality in pigs (Qiao et al., 2011; Han et al., 2017).

### T-cell immunoglobulin and mucin domain-1/4 and heat shock protein member 8

In 2020, our group demonstrated that PRRSV externalizes phosphatidylserine (PS) on the envelope as viral apoptotic mimicry, which is recognized by PS receptor TIM-1 or TIM-4 to trigger the downstream signaling pathway and macropinocytosis as an alternative entry pathway for PRRSV into MARC-145 cells and PAMs, respectively (Wei et al., 2020).

In this year, our group identified that HSPA8, a housekeeping chaperone, interacts with PRRSV GP4, and is involved in PRRSV attachment and internalization for the first time. Anti-HSPA8 polyclonal antibodies, inhibitors, and the recombinant soluble HSPA8 protein inhibit PRRSV infection *in vitro* (Wang et al., 2022).

Our findings enrich novel host cellular factors involved in PRRSV attachment and entry, and support them as potential antiviral targets against PRRSV infection.

### Syndecan-4 and epidermal growth factor receptor

Syndecans are a family of transmembrane HS proteoglycans and are involved in human cancers, infectious diseases, obesity, wound healing, and angiogenesis. In addition, syndecans act as receptors/co-receptors for viral infections (Fears and Woods, 2006). Wang et al. (2016a) found that syndecan-4 played a critical role in PRRSV attachment and entry in MARC-145 cells. They further found that syndecan-4 interacted with EGFR during PRRSV entry.

EGFR is a member of the ErbB family of receptor tyrosine kinases and a versatile signal transducer involved in various cellular processes. EGFR has also been exploited by various viruses during different stages (Carlin, 2021; Lai and Lee, 2022). Ni et al. (2015) and Wang et al. (2016b) demonstrated that EGFR was activated to initiate its downstream signal pathways, and modulated actin fragmentation and reorganization to facilitate PRRSV entry.

Based on their findings, we speculate that syndecan-4 functions as an alternative attachment factor, whereas EGFR is a signaling factor to mobilize the cytoskeleton for PRRSV entry. As its commercial targeted drugs are available, including inhibitors and mAbs (Yamaoka et al., 2017), EGFR may be a potential therapeutic target for controlling PRRSV infection.

### Sialic acid-binding Ig-like lectin-10

Xie et al. (2017) identified Siglec-10 as an alternative factor for PRRSV entry. They further stated preferential use of Sn or Siglec-10 by different PRRSV isolates (Xie et al., 2018). These studies suggest the utilization of several redundant Siglecs by PRRSV. The importance of Siglec-10 needs to be verified *in vivo* in the future.

## Perspectives and concluding remarks

It has long been acknowledged that host cellular factors involved in attachment and entry are the major determinants for PRRSV infection (Kreutz, 1998). In particular, CD163 is a well-documented indispensable receptor for PRRSV and, therefore, the optimal antiviral target (Su et al., 2021). CD163-targeted genetic editing, microRNAs, mAbs, inhibitors, proteins, and peptides support various potent antiviral tools against PRRSV. Despite the great successes of CD163-edited pigs in resistance to PRRSV, their clinical performances need continuous monitoring in consideration of the multifaceted functions of the receptor. In addition to CD163 and Sn, other reported host cellular factors require further *in vivo* validation of their involvement in PRRSV attachment and entry to evaluate whether they are qualified as antiviral targets. Moreover, it cannot be ruled out that there are unrecognized host cellular factors or even co-receptors for PRRSV attachment and entry.

PRRSV attachment and entry are initiated by the interactions between the viral envelope proteins and host cellular receptors/factors (Tian et al., 2012; Veit et al., 2014). However, it remains obscure which PRRSV envelope proteins are responsible for binding to the indispensable receptor CD163 (Das et al., 2010, 2011; Du et al., 2012; Wei et al., 2012). Our study showed that PRRSV GP5 was cleaved during viral membrane fusion (Hou et al., 2020). Whether it functions as a viral fusion protein needs further demonstration. As vaccination is another effective strategy to prevent and control PRRS, addressing these conundrums will clarify vaccine antigens and accelerate vaccine development (Stoian and Rowland, 2019).

The importance of antiviral drugs is increasingly apparent owing to PRRSV persistence in pigs. There are a growing number of antiviral agents reported interfering with PRRSV attachment or/and entry, including antimicrobial peptides protegrin-1 (Guo et al., 2015), cecropin P1 (Guo et al., 2014a), cecropin D (Liu et al., 2015), lavaspidic acid AB (Yang et al., 2013), glycyrrhizin (Duan et al., 2015), tetrahydroaltersolanol C (Zhang et al., 2016), curcumin (Du et al., 2017), griffithsin (Li et al., 2018a), iota-carrageenan (Guo et al., 2019b), polyethylenimine (Wang et al., 2019b), 25-hydroxycholesterol (*in vitro* and *in vivo*) (Ke et al., 2017; Song et al., 2017, 2019; Dong et al., 2018) and rottlerin (*in vitro* and *in vivo*) (Kang et al., 2021). Chinese herbal medicines also contain antiviral molecules to block PRRSV attachment and entry (Bello-Onaghise et al., 2020). Unfortunately, the specific targets of these antiviral agents have not yet been identified. A bigger concern is that the antiviral efficacies of the majority of these antiviral agents have not been validated *in vivo* unless otherwise stated. However, developing anti-PRRSV drugs remains a promising therapeutic strategy. It would be resultful to develop specific antiviral drugs blocking crucial host cellular factors, e.g., CD163, involved in PRRSV attachment and entry.

In conclusion, recent advances on host cellular factors involved in PRRSV attachment and entry have laid a strong basis for developing multi-target antiviral strategies. Intensive investigation in this field is still necessary to elucidate PRRSV infection, which is beneficial for preventing and controlling PRRS.

## Author contributions

RL recapitulated recent advances on host cellular factors involved in PRRSV attachment and entry and wrote the review. SQ and GZ provided guidance and modified the review. All authors contributed to the article and approved the submitted version.

## Conflict of interest

The authors declare that the research was conducted in the absence of any commercial or financial relationships that could be construed as a potential conflict of interest.

## Publisher’s note

All claims expressed in this article are solely those of the authors and do not necessarily represent those of their affiliated organizations, or those of the publisher, the editors and the reviewers. Any product that may be evaluated in this article, or claim that may be made by its manufacturer, is not guaranteed or endorsed by the publisher.

## Footnotes

1
https://talk.ictvonline.org/taxonomy/p/taxonomy-history?taxnode_id=201851832
